# 18S rDNA Sequences from Microeukaryotes Reveal Oil Indicators in Mangrove Sediment

**DOI:** 10.1371/journal.pone.0012437

**Published:** 2010-08-26

**Authors:** Henrique F. Santos, Juliano C. Cury, Flavia L. Carmo, Alexandre S. Rosado, Raquel S. Peixoto

**Affiliations:** Laboratory of Molecular Microbial Ecology, Department of General Microbiology, Institute of Microbiology Professor Paulo de Góes, Federal University of Rio de Janeiro (UFRJ), Rio de Janeiro, Brazil; University of Kansas, United States of America

## Abstract

**Background:**

Microeukaryotes are an effective indicator of the presence of environmental contaminants. However, the characterisation of these organisms by conventional tools is often inefficient, and recent molecular studies have revealed a great diversity of microeukaryotes. The full extent of this diversity is unknown, and therefore, the distribution, ecological role and responses to anthropogenic effects of microeukaryotes are rather obscure. The majority of oil from oceanic oil spills (e.g., the May 2010 accident in the Gulf of Mexico) converges on coastal ecosystems such as mangroves, which are threatened with worldwide disappearance, highlighting the need for efficient tools to indicate the presence of oil in these environments. However, no studies have used molecular methods to assess the effects of oil contamination in mangrove sediment on microeukaryotes as a group.

**Methodology/Principal Findings:**

We evaluated the population dynamics and the prevailing 18S rDNA phylotypes of microeukaryotes in mangrove sediment microcosms with and without oil contamination, using PCR/DGGE and clone libraries. We found that microeukaryotes are useful for monitoring oil contamination in mangroves. Our clone library analysis revealed a decrease in both diversity and species richness after contamination. The phylogenetic group that showed the greatest sensitivity to oil was the Nematoda. After contamination, a large increase in the abundance of the groups Bacillariophyta (diatoms) and Biosoecida was detected. The oil-contaminated samples were almost entirely dominated by organisms related to *Bacillariophyta* sp. and *Cafeteria minima*, which indicates that these groups are possible targets for biomonitoring oil in mangroves. The DGGE fingerprints also indicated shifts in microeukaryote profiles; specific band sequencing indicated the appearance of *Bacillariophyta* sp. only in contaminated samples and Nematoda only in non-contaminated sediment.

**Conclusions/Significance:**

We believe that the microeukaryotic targets indicated by our work will be of great applicability in biomonitoring hydrocarbons in mangroves under oil contamination risk or during recovery strategies.

## Introduction

Biomonitoring is defined as the systematic use of biological responses (biomarkers) to assess changes in the environment, which are often caused by anthropogenic effects [1]. Bioindicators are species, groups of species or biological communities whose presence, abundance and biological conditions are indicative of a particular environmental condition [2].

Microeukaryotes are probably the most abundant eukaryotes on Earth; they are found in all lakes and oceans [3]–[5] and are a subset of plankton known as microplankton, of which diatoms, dinoflagellates, coccolithophorids and a large number of protozoa are members. They are of vital importance to marine ecosystems (e.g. mangroves and salt marshes) because they represent the base of the pelagic food web in the ocean and changes in the composition and structure of this web can lead to profound changes at all trophic levels [6]. Mangrove sediment habitat is biologically rich and provides an unique ecological niche to a variety of organisms [7], which includes several microeukaryotic representatives [7]–[10].

This group can be an effective gauge in demonstrating the presence of contaminants because they exhibit the key features needed to be a good bioindicator, in particular their abundance, genetic diversity and reduced generation time, which allows for rapid responses to environmental changes [11]. Changes in the weather, regional geomorphic shifts and anthropogenic impacts on coastal areas establish the taxonomic characteristics and spatial-temporal dynamics of their communities [12], strengthening the great potential of these organisms as bioindicators of environmental changes, which was already described by conventional tools for filamentous fungi [13], yeast [10], [13], [14], nematodes [15], [16] and ciliates [17] in estuaries. Despite these findings, no studies have evaluated the impact of oil on microeukaryotes in mangrove sediments using molecular techniques.

In the last decade, the use of molecular techniques in microbial ecology has greatly increased our ability to identify microorganisms, in particular, prokaryotes, from various environments. In recent years, 18S rDNA clone libraries have been considered the gold standard approach for the development of molecular surveys of marine microbial diversity [18]–[20]. Several recent studies based on amplification and sequencing of the small subunit 18S ribosomal RNA gene fragment have revealed a great diversity of microeukaryotes [5], [21]–[24]. The full extent of this diversity is unknown, and therefore, it follows that their distribution, their patterns, their spatial and temporal dynamics, and their ecological role are rather obscure. For instance, mangroves are important and unique environments that are usually exposed to pollutants, such as those released by oil spills [25]–[27], and are considered by some authors as environments at risk of disappearance from the earth [28], [29]. Despite this, no studies have evaluated the impact of oil on microeukaryotes in mangrove sediment using molecular techniques.

In this study, we evaluated the impacts of oil on major microeukaryote groups in mangrove sediment by PCR/DGGE (Denaturing Gradient Gel Electrophoresis) and using clone libraries searching for potential candidates for use as bioindicators of oil or in further studies of mangrove bioremediation and biomonitoring using microeukaryotes.

## Methods

### Ethics Statement

The Institute of Microbiology Prof. Paulo de Góes and the Fundação Carlos Chagas Filho de Amparo à Pesquisa do Estado do Rio de Janeiro (FAPERJ) approved this study development.

### Sampling site and DNA extraction

The experiments were conducted in 288.5 cm^3^ PVC opaque tube microcosms (7.5×7 cm) that were open at the top and closed at bottom with PVC lids. Each microcosm received 350 g/L dry weight (195 cm^3^) of sediment from the Restinga da Marambaia, Rio de Janeiro, Brazil (23°3′27″ S 43°33′58″ W). The sediment (mud) sample comprised other ten sub-samples collected in a single location in the intertidal zone (20 cm deep). The sample was stored in a polyethylene bag that was transported to the laboratory, where the microcosms were immediately mounted (about 3 hours after sampling). Oil contamination of the microcosms was then performed (2% v/wt contamination). The oil (MF 380, the most transported oil in Rio de Janeiro) was mixed with the sediments to create homogeneous sediments that were shared among all microcosms. Triplicate samples were collected from the microcosms at different times (days): T0 (before oil contamination); T23 0% and 2% (23 days without oil and with 2% oil contamination); and T66 0% and 2% (66 days without oil and with 2% oil contamination). Every 2 days, 100 ml of distilled water was added to each microcosm to replace evaporated fluids. It was possible to observe a thin layer of water for ∼10 hours.

The 15 microcosms (triplicates for each condition [T0 (without oil), T23 (with and without oil) and T66 (with and without oil)] that were utilised and destroyed after each sampling time) were incubated in a greenhouse at room temperature (between 28–33°C).

For each microcosm sample, a 200 g sediment aliquot was taken for Total Petroleum Hydrocarbon (TPH) analyses. To assess the microeukaryotic communities associated with the sediment collected at different times and with different oil contamination levels, 0.5 g of the sediment from each microcosm replicate (for DGGE) or of a composed sample (mixed triplicates from each microcosm for each sampling time to perform the clone libraries) was used for DNA extraction using the Fast DNA Spin Kit for soil (QBIOgene, Carlsbad, CA) following the manufacturer's instructions. The extracted DNA was quantified using a Nanodrop ND-1000 spectrophotometer (Nanodrop Technologies, Wilmington DE). The integrity of the DNA extracted from the soil was confirmed by electrophoresis on a 0.8% agarose gel with 0.5× TBE buffer (45 mM Tris–borate, 1 mM EDTA, pH 8.0).

### Sediment evaluation of total petroleum hydrocarbon (TPH)

We used 7–10 replicates 10-g aliquots of (approximately 5 g dry) from each sample for extraction with a dichloromethane:acetone mixture (1∶1) in a Soxhlet extractor. Prior to the extraction, 100 ng of the standard p-terfenil-d14 was added to the sample to comprise the aromatic fraction. The volume of the raw extract was reduced in an evaporator with rotary flow of N_2_ to yield a volume of 1 ml. Fraction separation was accomplished by chromatography using a glass column, performed with silica/alumina.

The determination of total petroleum hydrocarbons was performed in a Varian Gas Cromatographer (GC) (CP 3800 MS Saturn 2200) equipped with a J&W (P/N 123–1334) DB-624 capillary column (30 m×0.32 mm I.D., 1.8 µm) according to EPA methods 8015 and 8030. The carrier gas was helium at a flow rate of 35 cm/sec, measured at 35°C. The initial temperature of the oven was 35°C, with an increase of 15°C/min (35–170°C). The split injector was set at 1∶40, and the injector temperature was set at 250°C. The injected volume was 1 µl. In the MSD detector, the detector temperature of the transfer line (full scan) was set at 280°C.

### PCR/DGGE

The amplification of specific fragments of the gene encoding the 18S ribosome subunit of the microeukaryotes was performed using the primers Ek7F-GC (ACCTGGTTGATCCTGCCAG-GC) and EK516R (ACCAGACTTGCCCTCC) [5], [22], [30] generating a product with about 500 bp. The amplification was performed in a solution containing 1× PCR buffer, 0.2 mM dNTP, 2.0 mM MgCl_2_, 0.75 U of recombinant Taq DNA polymerase (Promega), 10 ng of total DNA, 5 pmol of each primer and sterile Milli-Q water for a final volume of 25 µl. The reaction was performed in a thermocycler (Mastercycler Gradient, Eppendorf, Hamburg, Germany) under the following conditions: initial denaturation at 94°C for 30 s, 35 cycles at 94°C for 30 sec, 56°C for 45 sec and 72°C for 130 s with a final extension at 72°C for 7 min.

The amplicons were then separated by denaturing gradient gel electrophoresis (DGGE). The DGGE gels (30 to 65% of urea and formamide) were prepared with a solution of polyacrylamide (6%) in Tris-acetate (pH 8.3). The run was performed in 1× Tris-acetate-EDTA buffer at 60°C at a constant voltage of 75 V for 16 hours. The DGGE gels were stained with Sybr Green (Molecular Probes) and visualised using a Storm 860 Imaging System (GE Healthcare). The extracted gel fragment containing the bands was removed and treated with a QIAquick PCR Purification Kit (Qiagen) according to the manufacturer's instructions. Dendrograms were constructed after image capture and analysis by Pearson correlation coefficients (r), and cluster analysis was performed using the unweighted pair group method with average linkages (UPGMA), using BioNumerics software (Applied Maths, Ghent, Belgium). Each band was identified and its intensity was measured. This band intensity was then expressed as a proportion of the total intensity of all bands comprising a particular community profile.

### Clone Libraries

The 18S rRNA genes were amplified using the EK7F and EK516R primers^1,2^. Six PCR reactions (25 µl each) were performed with the followed mixture: 10 ng of DNA template, 1× PCR buffer, 0.2 mM dNTP, 2 mM MgCl_2_, 0.5 U of Taq DNA polymerase (Fermentas), 10 pmol of each primer and deionised water. PCR was performed using a thermocycler (Mastercycler, Eppendorf, Hamburg, Germany) under following conditions: initial denaturation at 95°C for 5 min, 30 cycles at 95°C for 1 min, 55°C for 1 min and 72°C for 1 min with a final extension at 72°C for 10 min.

Agarose gel electrophoresis of the 150 µl of PCR product was performed prior to purification and DNA was purified using the QIAquick Gel Extraction Kit (Qiagen) according to the manufacturer's instructions. Purified amplicons were ligated into the pGEM® T Easy Vector plasmid (Promega). The ligation products were transformed into DH5-α *Escherichia coli* competent cells. Positive clones were grown in LB medium and the extraction of plasmids was performed using the miniprep alkaline lysis method^20^. Sequencing of the insert was performed using the Big Dye Terminator system and an ABI-3730 automatic capillary sequencer (Applied Biosystems).

### Sequence Analysis

The electropherogram files generated by sequencing were processed using the Phred program [31] for base calling and trimming of vector and low-quality (<20) sequences. The high-quality sequences located between the rRNA primers were used for further analysis. Sequences were then aligned with ClustalX 1.81 [32]. The PHYLIP format output alignments were used to construct distance matrices within each library by using Dnadist from the phylip 3.6 package [33] with the default parameters and using the Jukes-Cantor model [34] option. The generated matrices were used as input files for DOTUR [35] to calculate the species richness using Chao1 [36] and ACE [37] estimators, rarefaction curves and the Shannon-Weaver diversity index [38]. The taxonomic affiliation was determined using the Blast program [39] through the web service provided by NCBI (http://www.ncbi.nlm.nih.gov/).

For the tree construction, one representative sequence of each OTU was randomly selected for use in the alignments. The nearest-neighbours sequences used for the construction of the previous trees were obtained using the selected representatives of each OTU and the Aligner tool through the web service provided by the SILVA database [40]. The FASTA file generated was edited for redundancy elimination, and the sequences were realigned and manually edited with the ClustalW aligner of the MEGA4.0 program [41]. Phylogenetic trees were constructed and edited using the MEGA 4.0 program with the neighbour-joining method, the Juke-Cantor model [34] option and a bootstrap value of 1000.

### Nucleotide sequence accession numbers

The sequences generated by clone libraries were deposited in the GenBank under the accession numbers HM228084-HM228385. The sequences generated by DGGE band excision were deposited in the GenBank under the accession numbers HM357130-HM357134.

## Results

The DGGE results indicated similarities above 95% between triplicates and above 90% between microcosm sediment samples without oil contamination from different sampling times, indicating that the microeukaryotic communities were stable in microcosms without oil disturbance during this period of time (66 days) (Data not shown). For this reason, we use T0 without oil as a representative sample of non-contaminated sediment to be the reference for changes caused by the presence of oil.

**Figure 1:**
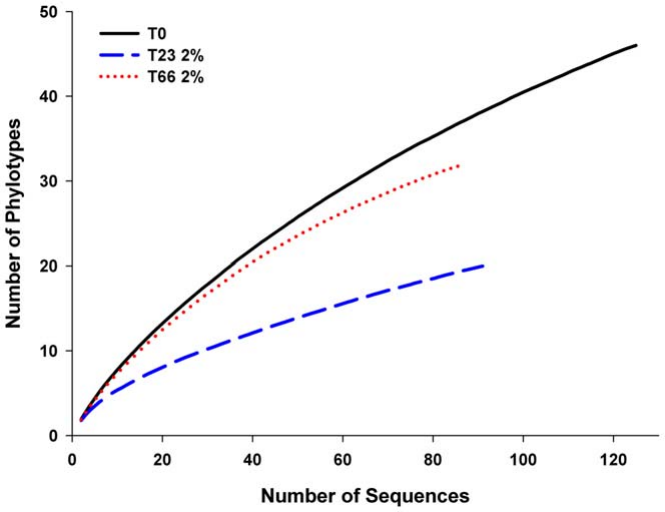
Rarefaction curves of partial sequences of 18S rDNA. The rarefaction curves from microcosm sediment samples were calculated by DOTUR_003_. T0, T23 2% and T66 2%: curves of 18S rDNA of each sampling T0, without oil contamination; T23 2%, 23 days after 2% of oil contamination; T66, 66 days after 2% of oil contamination.

The estimated values of OTU richness, diversity index, sample coverage and rarefaction curves are presented in Table 1 and Figure 1. The Shannon diversity indices for T23 2% and T66 2% are statistically lower than for T0 (T0 without oil contamination). Similar results are shown for the S_ACE_ and S_Chao1_ (Table 1).

**Table 1:** Estimated OTU richness, diversity indices and estimated sample coverage for 16S rRNA libraries of mangrove sediment samples.

Library	NS^a^	OTUs	OTUs richness estimators		Shannon^c^	ESC^d^
			ACE^b^	Chao1^b^		
0	125	46	97 (68; 164)	69 (55; 107)	319 (296; 342)	080
23	91	20	47 (28; 116)	37 (25; 84)	205 (177; 232)	087
60	87	32	51 (39; 86)	42 (35; 64)	289 (262; 316)	083

a Number of sequences for each library.

b Calculated with DOTUR at the 3% distance level.

c Shannon diversity index calculated using DOTUR (3% distance).

d Estimated sample coverage: Cx = 1−(Nx/n), where Nx is the number of unique sequences and n is the total number of sequences.

Values in brackets are 95% confidence intervals as calculated by DOTUR.

**Figure 2:**
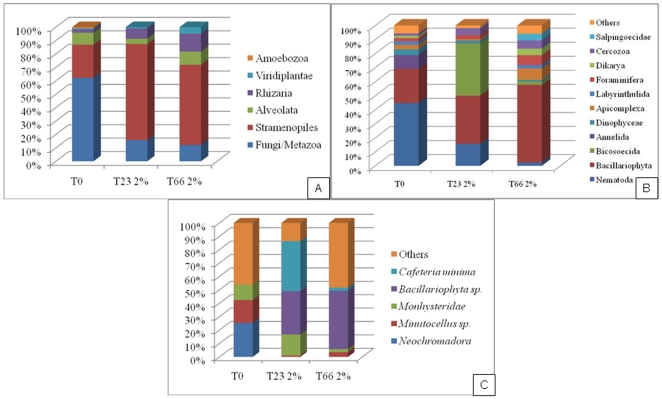
Distribution of partial sequences of the eukaryotic 18S rRNA gene from mangrove sediment. Affiliation was performed using NCBI-Blast searches A: Taxonomic groups B: Phyla C: Genus/species T0, without oil contamination; T23 2%, 23 days after 2% of oil contamination; T66, 66 days after 2% of oil contamination.

We observed a great predominance of the Fungi/Metazoa group, corresponding to about 70% of all sequences obtained from sediment without oil, followed by Stramenopiles (25%), Alveolata (9%), Rhizaria (4%) and Viridiplantae, Amoebozoa and Ichthyosporea (1%) (Figure 2a and Table S1). The dominant phyla observed were Nematoda and Bacillariophyta, at 45% and 24% (Figure 2b and Table S1), respectively, and the dominant species were related to the genera *Monhysteridae*, *Minutocellusnd* and, *e*specially *Neochromadora*, corresponding to about 55% of the whole microeukaryote diversity from the Restinga da Marambaia sediment (Figure 2c and Table S1).

The dominant group that proved sensitive to oil contamination was the Fungi/Metazoa; the oil treatment reduced their relative abundance to only 16% and 11% of the sequences, 23 and 66 days after oil contamination respectively. The Stramenopiles became the dominant taxonomic group, increasing from 25% at T0 to 71% 23 days after oil contamination and to 61% after 66 days (Figure 2a). Interestingly, the Bacillariophyta phylum demonstrated a great abundance before and after oil contamination, showing a gradual increase after oil contamination while Nematoda decrease gradually after exposure (Figure 2b). The Bicosoecida phylum was only detectable after oil contamination and was abundantly present only 23 days after contamination (Figure 2b).

Looking at the representatives of the dominant taxonomic group Bacillariophyta, there was an increase of sequences from the genus Bacillariophyta sp. after oil contamination, from zero in non-contaminated sediment to 32.4% and 43.5% of all sequences, 23 and 66 days after oil application (Figure 2c and Table S1). Representing the Bisoecida group, the *Cafeteria minima* species, belonging to Stramenopila taxonomic group, was the dominant species 23 days after oil contamination and showed a curious profile; 66 days after oil exposure, this species represented only 2.3% of the microeukaryote community. Despite this observation, the TPH levels from T23 and T66 were similar (Figure S1).

The most oil-sensitive species were members of the *Neochromadora* and *Minutocellus* genera and decreased to between 0–1% after oil contamination (Figure 2c and Table S1).

**Figure 3:**
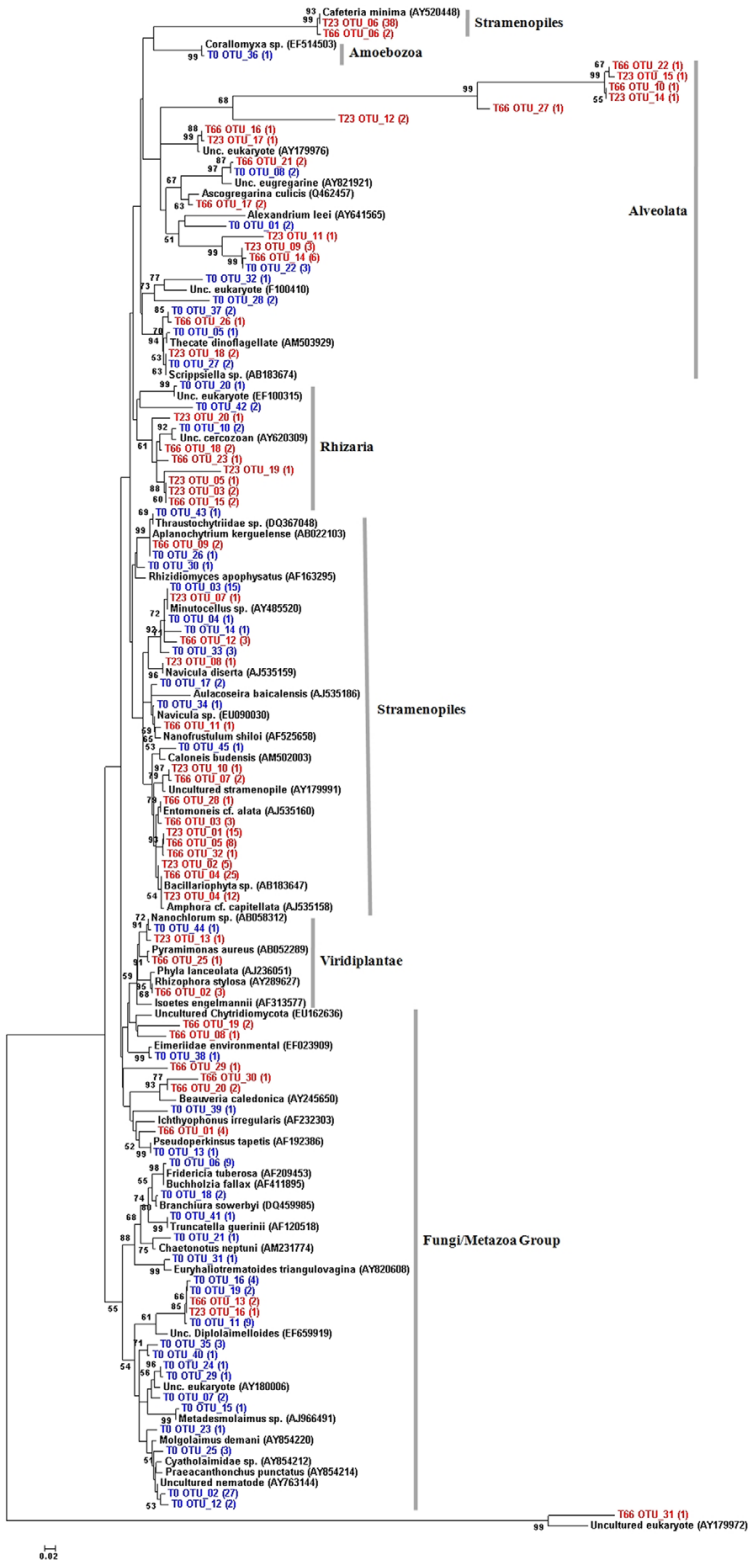
Phylogram of the microeukaryotic 18S rRNA phylotypes obtained from the microcosm sediment samples. A representative sequence of each OTU determined by DOTUR_003_ and the nearest neighbours obtained by using the aligner tool of the SILVA database project were used OTUs nomination: T0, without oil contamination (in blue); T23 2%, 23 days after 2% of oil contamination (in red); T66, 66 days after 2% of oil contamination (in red) The phylogram was calculated with MEGA 40 using the neighbour-joining method and the Jukes-Cantor model Numbers at the branches show the bootstrap percentages (above 50% only) after 1000 replications of bootstrap sampling.

A phylogenetic tree was constructed (Figure 3) by combining the phylogenetic affiliations of the obtained sequences from the three samples studied and the most similar sequences found in NCBI. We found that a great predominance of samples from the T0 sample grouped into the Fungi/Metazoa cluster. Concerning the Stramenopila group, we observe the clustering of *Bacillariophyta sp*. sequences with OTUs (Operational Taxonomic Units) from contaminated samples only. The Amoebozoa group is represented by only one OTU from T0, while the groups Rhizaria, Viridiplantae and Alveolata presented sequences from the three studied samples.

**Figure 4:**
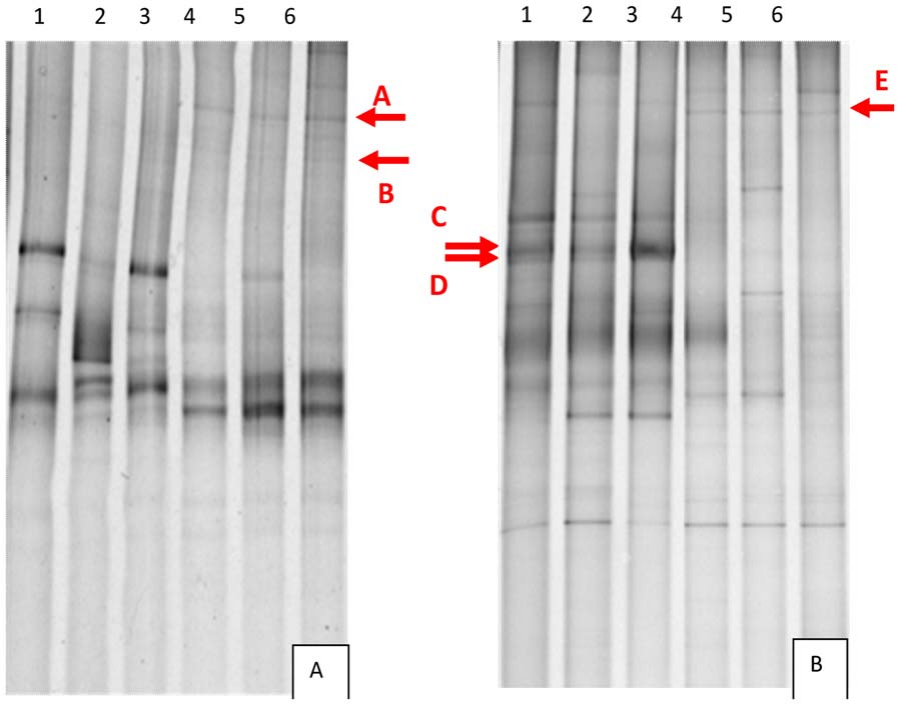
Sybr green-stained DGGE (30 to 65% of urea and formamide) gels of 18S rDNA fragments. A: 1, T0a; 2, T0b; 3, T0c (replicates); 4, T23 2% a (23 days after 2% of oil contamination); 5, T23 2% b; 6, T23 2% c (replicates) B: 1, T0a; 2, T0b; 3, T0c (replicates); 4, T66 2% a (66 days after 2% of oil contamination); 5, T66 2% b; 6, T66 2% c (replicates).

The DGGE analysis also demonstrated shifts in the microeukaryote profiles in sediment samples with the addition of oil. As demonstrated by clone libraries, some extracted gel bands from sediment samples 23 days after oil contamination also indicated the appearance of Bacillariophyta, specifically the algae species *Amphora montana*, with 96% similarity (Figure 4a and Table 2). In sediment samples evaluated 66 days after oil contamination, a band fragment related to the Alveolata taxonomic group was detected, while in samples without oil, bands containing DNA fragments related to Nematoda representatives were observed (Figure 4b and Table 2).

**Table 2:** Phylogenetic affiliations of microeukaryotes in DGGE-extracted band sequences.

*Bands*	*Phylogenetic affiliation*	*Most similar Specie or strain (access number)*	*Similarity(%)*	*Source*
A	**Stramenopiles**Bacillariophyta	*Amphora Montana* (AJ243061)	96%	Algae from marine environment
B	**Stramenopiles**Bacillariophyta	*Amphora sp*(AB183590)	97%	Algae from marine environment
C	**Metazoa**Nematoda	*Laxus oneistus*(Y16919)	87%	Sand of coral reefs in Belize
D	**Metazoa**Nematoda	Diplolaimelloides environmental sample(EF659926)	79%	Saline environments
E	**Alveolata**Ciliophora	Ciliado não cultivado(DQ115950)	74%	PAH-contaminated soil

## Discussion

The use of microeukaryotes as bioindicators of environmental shifts has been proposed by some authors, but no studies were available that considered the application of molecular tools to evaluate the effects of oil on microeukaryotes in mangrove sediment. Our clone library results, which indicated a predominance of the Fungi/Metazoa group (∼70%) followed by Stramenopiles (25%) and Alveolata (9%) (Figure 2a), were specific to the studied mangrove when compared to other environments described in the literature, suggesting that this diversity pattern is likely related to the mangrove environment. For instance, a predominance of the groups Alveolata and Stramenopiles (41% and 28%, respectively) was observed in the water of the Mariager Fjord, Denmark [42]. In deep sea sediments rich in methane in Sagami Bay, Japan, a predominance of Fungi (63.2%), entirely represented by *Cryptococcus curvatus*, was observed, followed by Alveolata (15%), Cercozoa (8.6%) and Stramenopiles (5.4%) [43]. In a water reservoir in an abandoned pyrite mine in Portugal, which features extremely low pH and high concentrations of heavy metals, 54.8% of the microeukaryote sequences belonged to two clones of the Viridiplantae group and 14.6% to a clone of Stramenopiles [44]. Clones belonging to the Fungi and Alveolata groups were also detected [44]. Cheung and colleagues [21] evaluated microeukaryotic diversity in coastal waters by pyrosequencing and described Stramenopiles, dinoflagellates, ciliates and prasinophytes as the dominant groups, comprising approximately 27%, 19%, 11% and 11% of the total population, respectively.

Concerning the mangrove ecosystem, some microeukatyotic indicators were already suggested [10]. For instance, it was previously described by using cultivation methods that filamentous fungi and yeasts were also significantly (P <0.05) lower in polluted United Arab Emirates mangrove sediment when compared to non-polluted sediment [13]. The species *Yarrowia lipolytica* (anamorph *Candida lipolytica*) is typically a very strong assimilator of hydrocarbons and has been suggested as an indicator of oil contamination in marine and estuarine environments [10]. A number of *Trichosporon* species, which was found in our samples, are common in aquatic sediments and polluted waters, and are able to assimilate phenolic compounds [45]. There is also a suggestion of using the yeast *Kluyveromyces estuarii* as an oil indicator in mangroves [10]. Another conventional study demonstrated that ciliates could serve as good bioindicators in assessing the qualities of organically polluted mangrove sediment [17].

The ACE and Chao1 richness estimators and the Shannon diversity index indicated a decrease in microeukaryotic diversity and species richness after contamination, and the phylogenetic group that showed the greatest sensitivity to oil was Nematoda (nematodes). Corroborating clone library results, DGGE band fragments related to Nematoda were only detected in non-contaminated samples. Moreno and colleagues [15] drew attention to the fact that nematodes offer a promising possibility for assessing changes in community structure, due to their high structural and functional diversity. Nematodes have been used in biomonitoring studies and are suitable indicators of the impacts of pollution on marine ecosystems [16], [46]. Beyrem and colleagues [40] investigated the effects of lubricant oils on marine Nematoda populations and observed that total nematode abundance, species richness and species number decreased significantly in all lubricant-contaminated microcosms. These authors indicated that the study of nematode populations is a useful tool for assessing the environmental quality of impacted ecosystems and for identifying vulnerable areas on which management actions should first be focused. Our results indicate that a similar conclusion can be applied to oil-contaminated mangrove monitoring.

After contamination, it was also possible to detect a large increase in the abundance of the group Bacillariophyta (diatoms), which was already a part of the dominant community in native sediment. Bicosoecida were also detected, with the oil contaminated samples almost entirely dominated by *Bacillariophyta* sp. at both times after oil contamination, and *Cafeteria minima* was present 23 days after oil contamination. *C. minima* demonstrated a curious profile, being dominant 23 days after oil contamination and decreasing substantially 66 days after the oil spill. This decrease was not related to the TPH (Total Petroleum Hydrocarbons) level observed because the total concentrations were similar at the two sampling times after oil contamination. A plausible explanation is that the fraction of available oil may be different during sampling times, or competition with other organisms may have taken place 66 days after the addition of oil.

The Bacillariophyta group was previously described as a dominant member of mangrove microeukaryotic communities [47], and belongs to the Stramenopile or Heterokonta rank, which contain key oceanic algal classes (e.g., the ubiquitous diatoms) and heterotrophic groups such as the Bicosoecids [5]. As described above, the Bicosoecid *C. minima*, which was not detected before oil contamination, represented almost 40% of all sequences 23 days after oil contamination. Some studies, such as a recent investigation in the Nile River of Egypt, have reported the presence of Bacillariophyta in environments contaminated with oil, with this group being more abundant at a site contaminated by oil [48]. Cesar [49] also observed that there was an increase of some species of diatoms after a spill of diesel and vegetable oil in the Rio Negro, Paraná. Our DGGE results corroborated clone libraries, with bands that were specific to oil-contaminated samples and that presented similarities to sequences of Bacillariophyta, specifically the algae genus *Amphora*.

Molecular evaluation of microeukaryotic communities in environmental samples is becoming an increasingly studied subject, indicating that the diversity of this group is higher than previously described [5], [50], Indeed, very little is known about such diversity in many ecosystems. For instance, despite the large number of reports of the presence of Bacillariophyta in oil-impacted areas, a large number of the oil microcosm sequences reported in this work have not been described previously in areas with petroleum hydrocarbon contamination.

In conclusion, the use of molecular techniques for monitoring microeukaryotic communities in oil-impacted mangroves has emerged as a potential tool to quickly and efficiently indicate anthropogenic effects. *Bacillariophyta sp*. and *Cafeteria minima* are promissing targets for the biomonitoring of the presence of petroleum hydrocarbons in mangrove sediments, with Nematoda being a very sensitive group, and *Neochromadora* and *Minutocellus* as the most sensitive genera.

Despite the fact that the Restinga da Marambaia mangrove (the origin of the sediment used in our microcosm construction) is an environment with no history of contamination by petroleum hydrocarbons, a survey of the native microeukaryotic community of this area and assays to evaluate possible impacts of oil on the environment are of extreme importance. This area is located in Sepetiba Bay, where the Itaguaí Port is expanding to accommodate larger ships, which makes this preserved marine environment highly susceptible to an ecological disaster caused by oil spills. Other environments under risk or under active contamination can also be monitored to prevent oil effects and during the recovery process. Despite the possible, and expected, variability of microbial diversity in different mangroves, we believe that the microeukaryotes indicated in this study will be useful for monitoring oil contamination in these environments.

As an example of a practical application, we suggest the use of such groups as targets to be used in qPCR experiments to quantify the abundance of these groups in distinct mangroves contaminated with oil. This would permit evaluation of the presence and abundance of these indicators in mangroves with different levels of oil, which could validate this approach as useful in field monitoring. Also, based on our study, further works using molecular, isolation and microscopic techniques can provide more detailed information concerning species with enhanced sensitivity or tolerance to oil contamination.

## Supporting Information

Table S1 Closest relative sequences obtained with NCBI-Blast search using generated partial 18S rRNA sequences from microcosm sediment samples.
(0.19 MB DOC)

Figure S1 Total Petroleum Hydrocarbons (TPH) concentrations during experiment sampling. T0, without oil contamination; T23 2%, 23 days after 2% of oil contamination; T66, 66 days after 2% of oil contamination (duplicates).
(9.47 MB TIF)
