# Supplementary material for: 18S rDNA Sequences from Microeukaryotes Reveal Oil Indicators in Mangrove Sediment
Source: PLoS One. 2010 Aug 26;5(8):e12437. doi: 10.1371/journal.pone.0012437 (PMC2928742; doi:10.1371/journal.pone.0012437)
Supplement: Table S1 — Closest relative sequences obtained with NCBI-Blast search using generated partial 18S rRNA sequences from microcosm sediment samples. (0.19 MB DOC) [file pone.0012437.s001.doc]

**Table S1: Closest relative sequences obtained with NCBI-Blast search using generated partial 18S rRNA sequences from microcosm sediment samples.**

| ***Phylogenetic affiliation*** | ***Closest described relative***  ***Species or strain (accession no.)*** | | ***Closest phylotype***  ***Sequence (accession no.)*** | | | |  |  | ***Sample*** |  | **OTU** |
| --- | --- | --- | --- | --- | --- | --- | --- | --- | --- | --- | --- |
|  |  | ***Sim. (%)*** |  |  | ***Sim. (%)*** |  |  | **T0 0%** | **T23 2%** | **T66 2%** |  |
| **Fungi/Metazoa** |  |  |  |  |  |  |  |  |  |  |  |
| Ichthyophonida | Pseudoperkinsus tapetis (AF192386) | 99 |  | Pseudoperkinsus tapetis (AF192386) | 99 |  |  | 0,8 | - | - | T0_13 |
|  | Dermocystidium sp.( [AF533950.1](http://www.ncbi.nlm.nih.gov/entrez/query.fcgi?cmd=Retrieve&db=Nucleotide&list_uids=28629211&dopt=GenBank&RID=NB95VVTG01S&log$=nucltop&blast_rank=1)) | 83 |  | Dermocystidium sp.( [AF533950.1](http://www.ncbi.nlm.nih.gov/entrez/query.fcgi?cmd=Retrieve&db=Nucleotide&list_uids=28629211&dopt=GenBank&RID=NB95VVTG01S&log$=nucltop&blast_rank=1)) | 83 |  |  | 1 | - | 1,2 | T66_29 |
| Choanoflagellida | Salpingoeca urceolata ([EU011931.1](http://www.ncbi.nlm.nih.gov/entrez/query.fcgi?cmd=Retrieve&db=Nucleotide&list_uids=157780195&dopt=GenBank&RID=NB95VVTG01S&log$=nucltop&blast_rank=1)) | 93 |  | Salpingoeca urceolata ([EU011931.1](http://www.ncbi.nlm.nih.gov/entrez/query.fcgi?cmd=Retrieve&db=Nucleotide&list_uids=157780195&dopt=GenBank&RID=NB95VVTG01S&log$=nucltop&blast_rank=1)) | 93 |  |  | - | - | 4,7 | T66_1 |
| Fungi |  |  |  |  |  |  |  |  |  |  |  |
| Dikarya | Melanops tulasnei (FJ824761) | 83 |  | Uncultured eukaryote |  |  |  | 1,6 | - | - | T0_28 |
|  | Graphiola phoenicis (GRAJCMA) | 83 |  | Uncultured eukaryote (EF100410) |  |  |  | 0,8 | - | - | T0_32 |
|  | Trichosporon lignicola([DQ836003.2](http://www.ncbi.nlm.nih.gov/entrez/query.fcgi?cmd=Retrieve&db=Nucleotide&list_uids=111999559&dopt=GenBank&RID=NB95VVTG01S&log$=nucltop&blast_rank=1)) | 90 |  | Trichosporon lignicola([DQ836003.2](http://www.ncbi.nlm.nih.gov/entrez/query.fcgi?cmd=Retrieve&db=Nucleotide&list_uids=111999559&dopt=GenBank&RID=NB95VVTG01S&log$=nucltop&blast_rank=1)) | 90 |  |  | - | - | 1,2 | T66_8 |
|  | Eutypa lata. ([DQ836896.1](http://www.ncbi.nlm.nih.gov/entrez/query.fcgi?cmd=Retrieve&db=Nucleotide&list_uids=110810428&dopt=GenBank&RID=NB95VVTG01S&log$=nucltop&blast_rank=1)) | 99 |  | Eutypa lata ([DQ836896.1](http://www.ncbi.nlm.nih.gov/entrez/query.fcgi?cmd=Retrieve&db=Nucleotide&list_uids=110810428&dopt=GenBank&RID=NB95VVTG01S&log$=nucltop&blast_rank=1)) | 99 |  |  | - | - | 2,35 | T66_20 |
|  | Sordariomycetes sp.( [FJ477277.1](http://www.ncbi.nlm.nih.gov/entrez/query.fcgi?cmd=Retrieve&db=Nucleotide&list_uids=224925898&dopt=GenBank&RID=NB95VVTG01S&log$=nucltop&blast_rank=1)) | 93 |  | Sordariomycetes sp.( [FJ477277.1](http://www.ncbi.nlm.nih.gov/entrez/query.fcgi?cmd=Retrieve&db=Nucleotide&list_uids=224925898&dopt=GenBank&RID=NB95VVTG01S&log$=nucltop&blast_rank=1)) | 93 |  |  | - | - | 1,2 | T66_30 |
|  |  |  |  |  |  |  |  |  |  |  |  |
| Metazoa |  |  |  |  |  |  |  |  |  |  |  |
| Nematoda | Paracyatholaimus intermedius (FJ969133) | 97 |  | Paracyatholaimus intermedius (FJ969133) | 97 |  |  | 1,6 | - | - | T0_12 |
|  | Monhysteridae environmental sample([GQ921338.1](http://www.ncbi.nlm.nih.gov/entrez/query.fcgi?cmd=Retrieve&db=Nucleotide&list_uids=267798852&dopt=GenBank&RID=NBGS2J0201S&log$=nucltop&blast_rank=1)) | 94-95 |  | Monhysteridae environmental sample([GQ921338.1](http://www.ncbi.nlm.nih.gov/entrez/query.fcgi?cmd=Retrieve&db=Nucleotide&list_uids=267798852&dopt=GenBank&RID=NBGS2J0201S&log$=nucltop&blast_rank=1)) | 94-95 |  |  | 11,5 | 15,7 | 2,35 | T0_11,16,19; T23_16; T66_13 |
|  | Daptonema normandicum (AY854224) | 91-92 |  | Uncultured eukaryote (AY180006) | 92-93 |  |  | 3,3 | - | - | T0_7,24,  29 |
|  | Calomicrolaimus sp.( [AY854219.1](http://www.ncbi.nlm.nih.gov/entrez/query.fcgi?cmd=Retrieve&db=Nucleotide&list_uids=62082928&dopt=GenBank&RID=NBPBMNWY013&log$=nucltop&blast_rank=4)) |  |  | Uncultured eukaryote([GU072521.1](http://www.ncbi.nlm.nih.gov/entrez/query.fcgi?cmd=Retrieve&db=Nucleotide&list_uids=262358020&dopt=GenBank&RID=NBPBMNWY013&log$=nucltop&blast_rank=1)) |  |  |  | 0,8 | - | - | T0_23 |
|  | Ptycholaimellus sp. (FJ040472) | 99 |  | Ptycholaimellus sp. (FJ040472) | 99 |  |  | 2,5 | - | - | T0_25 |
|  | Neochromadora (AY854210) | 92-97 |  | Neochromadora (AY854210) | 92-97 |  |  | 25,4 | - | - | T0_2,35,39 |
| Annelida | Grania variochaeta (AY365459) | 91 |  | Uncultured eukaryote ([FN393344.1](http://www.ncbi.nlm.nih.gov/entrez/query.fcgi?cmd=Retrieve&db=Nucleotide&list_uids=261259740&dopt=GenBank&RID=NBPBMNWY013&log$=nucltop&blast_rank=1)) | 92 |  |  | 7,3 | - | - | T0_6 |
|  | Heterodrilus minisetosus (AF411885) | 98 |  | Heterodrilus minisetosus (AF411885) | 98 |  |  | 1,6 | - | - | T0_18 |
|  | Daptonema setosum (AY854226) | 98 |  | Daptonema setosum (AY854226) | 98 |  |  | 0,8 | - | - | T0_15 |
| Platyhelminthes | Pseudohaliotrema sphincteroporus (AJ287568) | 92 |  | Pseudohaliotrema sphincteroporus (AJ287568) | 92 |  |  | 0,8 | - | - | T0_31 |
| Gastrotricha | Chaetonotus neptuni (AM231774) | 94 |  | Chaetonotus neptuni (AM231774) | 94 |  |  | 0,8 | - | - | T0_21 |
| Mollusca | Ilyanassa obsoleta (AY145379) | 97 |  | Ilyanassa obsoleta (AY145379) | 97 |  |  | 0,8 | - | - | T0_41 |
| [**Stramenopiles**](http://www.ncbi.nlm.nih.gov/Taxonomy/Browser/wwwtax.cgi?mode=Undef&id=33634&lvl=3&keep=1&srchmode=1&unlock) |  |  |  |  |  |  |  |  |  |  |  |
| Bacillariophyta | Licmophora flabellata (EF423409) | 89 |  | Uncultured marine eukaryote (EF527077) | 99 |  |  | 0,8 | - | - | T0_30 |
|  | cf. Minutocellus sp. (AY485520) | 88-97 |  | cf. Minutocellus sp. (AY485520) | 88-100 |  |  | 17,2 | 1 | 3,5 | T0_3,4,14,39; T23_7; T66_12 |
|  | Navicula tripunctata (AM502028) | 96 |  | Uncultured stramenopile (AY179995) | 98 |  |  | 1,6 | - | - | T0_17 |
|  | Aplanochytrium sp. (DQ367045) | 100 |  | Aplanochytrium sp. (DQ367045) | 100 |  |  | 0,8 | - | - | T0_26 |
|  | Nitzschia longíssima (AY881968) | 99 |  | Nitzschia longíssima (AY881968) | 99 |  |  | 0,8 | - | - | T0_34 |
|  | Pinnularia rupestris (AM501992) | 93 |  | Pinnularia rupestris (AM501992) | 93 |  |  | 0,8 | - | - | T0_45 |
|  | Bacillariophyta sp. ([AB183647.1](http://www.ncbi.nlm.nih.gov/entrez/query.fcgi?cmd=Retrieve&db=Nucleotide&list_uids=63148783&dopt=GenBank&RID=1SUWVUZ2016&log$=nucltop&blast_rank=1)) | 92-100 |  | Bacillariophyta sp. ([AB183647.1](http://www.ncbi.nlm.nih.gov/entrez/query.fcgi?cmd=Retrieve&db=Nucleotide&list_uids=63148783&dopt=GenBank&RID=1SUWVUZ2016&log$=nucltop&blast_rank=1)) | 92-100 |  |  | - | 32,4 | 43,5 | T23_1,2,4,10; T66_4,5,7,9 |
|  | Navicula diserta  ([AJ535159.1](http://www.ncbi.nlm.nih.gov/entrez/query.fcgi?cmd=Retrieve&db=Nucleotide&list_uids=41393012&dopt=GenBank&RID=NB61GNHA01N&log$=nucltop&blast_rank=1)) | 98 |  | Navicula diserta  ([AJ535159.1](http://www.ncbi.nlm.nih.gov/entrez/query.fcgi?cmd=Retrieve&db=Nucleotide&list_uids=41393012&dopt=GenBank&RID=NB61GNHA01N&log$=nucltop&blast_rank=1)) | 98 |  |  | - | 1 | - | T23_8 |
|  | Staurosira sp. ([EF465491.1](http://www.ncbi.nlm.nih.gov/entrez/query.fcgi?cmd=Retrieve&db=Nucleotide&list_uids=149127105&dopt=GenBank&RID=NB95VVTG01S&log$=nucltop&blast_rank=1)) | 97 |  | Staurosira sp. ([EF465491.1](http://www.ncbi.nlm.nih.gov/entrez/query.fcgi?cmd=Retrieve&db=Nucleotide&list_uids=149127105&dopt=GenBank&RID=NB95VVTG01S&log$=nucltop&blast_rank=1)) | 97 |  |  | - | - | 1,2 | T66_11 |
|  | Entomoneis cf. alata ([AJ535160.1](http://www.ncbi.nlm.nih.gov/entrez/query.fcgi?cmd=Retrieve&db=Nucleotide&list_uids=41393013&dopt=GenBank&RID=NB95VVTG01S&log$=nucltop&blast_rank=1)) | 97-98 |  | Entomoneis cf. alata ([AJ535160.1](http://www.ncbi.nlm.nih.gov/entrez/query.fcgi?cmd=Retrieve&db=Nucleotide&list_uids=41393013&dopt=GenBank&RID=NB95VVTG01S&log$=nucltop&blast_rank=1)) | 97-98 |  |  | - | - | 4,7 | T66_3, 28 |
|  | Amphora Montana([AJ243061.1](http://www.ncbi.nlm.nih.gov/entrez/query.fcgi?cmd=Retrieve&db=Nucleotide&list_uids=5101678&dopt=GenBank&RID=NB95VVTG01S&log$=nucltop&blast_rank=1)) | 93-94 |  | Amphora Montana([AJ243061.1](http://www.ncbi.nlm.nih.gov/entrez/query.fcgi?cmd=Retrieve&db=Nucleotide&list_uids=5101678&dopt=GenBank&RID=NB95VVTG01S&log$=nucltop&blast_rank=1)) | 93-94 |  |  | - | - | 2,35 | T66_31,32 |
| Bicosoecida | Cafeteria minima  ([AY520449.1](http://www.ncbi.nlm.nih.gov/entrez/query.fcgi?cmd=Retrieve&db=Nucleotide&list_uids=48762854&dopt=GenBank&RID=NB61GNHA01N&log$=nucltop&blast_rank=1)) | 98-99 |  | Cafeteria minima  ([AY520449.1](http://www.ncbi.nlm.nih.gov/entrez/query.fcgi?cmd=Retrieve&db=Nucleotide&list_uids=48762854&dopt=GenBank&RID=NB61GNHA01N&log$=nucltop&blast_rank=1)) | 98-99 |  |  | - | 37,3 | 2,35 | T23_6; T66_6 |
| Labyrinthulida | Oblongichytrium sp. (FJ799795) | 81 |  | Oblongichytrium sp. (FJ799795) | 81 |  |  | 1,6 | - | - | T0_42 |
|  | Thraustochytriidae sp. (DQ367048) | 98 |  | Thraustochytriidae sp. (DQ367048) | 98 |  |  | 0,8 | - | - | T0_43 |
|  | Aplanochytrium stocchinoi([AJ519935.1](http://www.ncbi.nlm.nih.gov/entrez/query.fcgi?cmd=Retrieve&db=Nucleotide&list_uids=30268157&dopt=GenBank&RID=NBY0BSNX01N&log$=nucltop&blast_rank=5)) | 99 |  | Uncultured labyrinthulid([FJ800649.1](http://www.ncbi.nlm.nih.gov/entrez/query.fcgi?cmd=Retrieve&db=Nucleotide&list_uids=225545995&dopt=GenBank&RID=NBY0BSNX01N&log$=nucltop&blast_rank=1)) | 100 |  |  | - | - | 2,35 | T66_9 |
| **Alveolata** |  |  |  |  |  |  |  |  |  |  |  |
| Dinophyceae | Thecate dinoflagellate (AM503929) | 99 |  | Thecate dinoflagellate (AM503929) | 99 |  |  | 0,8 | - | - | T0_5 |
|  | Scrippsiella sp. (AB183677) | 99-100 |  | Scrippsiella sp. (AB183677) | 99-100 |  |  | 1,6 | 1,9 | - | T0_27; T23_18 |
|  | Warnowia sp. (FJ947040) | 97 |  | Uncultured eukaryote (AY664893) | 98 |  |  | 1,6 | - | - | T0_37; T66_26 |
|  | Warnowia sp.( [FJ947040.1](http://www.ncbi.nlm.nih.gov/entrez/query.fcgi?cmd=Retrieve&db=Nucleotide&list_uids=239775419&dopt=GenBank&RID=NB95VVTG01S&log$=nucltop&blast_rank=3)) | 98 |  | Warnowia sp.( [FJ947040.1](http://www.ncbi.nlm.nih.gov/entrez/query.fcgi?cmd=Retrieve&db=Nucleotide&list_uids=239775419&dopt=GenBank&RID=NB95VVTG01S&log$=nucltop&blast_rank=3)) | 98 |  |  | - | - | 1,2 |  |
| Ciliophora | Varistrombidium sp. (DQ811090) | 97 |  | Uncultured eukaryote (EU371395) | 98 |  |  | 0,8 | - | - | T0_20 |
| Apicomplexa | Eimeriidae (EF023909) | 98 |  | Eimeriidae (EF023909) | 98 |  |  | 0,8 | - | - | T0_38 |
|  | Selenidium orientale(FJ832161) | 80 |  | Selenidium orientale(FJ832161) | 80 |  |  | 1,6 | - | - | T0_1 |
|  | Cryptosporidiidae (EF024471) | 91 |  | Cryptosporidiidae (EF024471) | 91 |  |  | 1,6 | - | 2,35 | T0_8; T66_21 |
|  | Eimeriidae environmental ([EF024503.1](http://www.ncbi.nlm.nih.gov/entrez/query.fcgi?cmd=Retrieve&db=Nucleotide&list_uids=166083980&dopt=GenBank&RID=NB95VVTG01S&log$=nucltop&blast_rank=1)) | 88 |  | Eimeriidae environmental ([EF024503.1](http://www.ncbi.nlm.nih.gov/entrez/query.fcgi?cmd=Retrieve&db=Nucleotide&list_uids=166083980&dopt=GenBank&RID=NB95VVTG01S&log$=nucltop&blast_rank=1)) | 88 |  |  | - | - | 2,35 | T66_19 |
|  | Babesia felis ([AY452698.1](http://www.ncbi.nlm.nih.gov/entrez/query.fcgi?cmd=Retrieve&db=Nucleotide&list_uids=42556120&dopt=GenBank&RID=NB61GNHA01N&log$=nucltop&blast_rank=11)) | 82 |  | Uncultured eukaryote ([AY179976.1](http://www.ncbi.nlm.nih.gov/entrez/query.fcgi?cmd=Retrieve&db=Nucleotide&list_uids=27802552&dopt=GenBank&RID=NB61GNHA01N&log$=nucltop&blast_rank=1)) | 95-96 |  |  | - | 1 | 1,2 | T23_17; T66_16 |
|  | Ascogregarina culicis([DQ462457.1](http://www.ncbi.nlm.nih.gov/entrez/query.fcgi?cmd=Retrieve&db=Nucleotide&list_uids=92430153&dopt=GenBank&RID=NB95VVTG01S&log$=nucltop&blast_rank=1)) | 91 |  | Ascogregarina culicis([DQ462457.1](http://www.ncbi.nlm.nih.gov/entrez/query.fcgi?cmd=Retrieve&db=Nucleotide&list_uids=92430153&dopt=GenBank&RID=NB95VVTG01S&log$=nucltop&blast_rank=1) | 91 |  |  |  |  | 2,35 | T66_17 |
| Perkinsea | Perkinsus sp ([AF042708.1](http://www.ncbi.nlm.nih.gov/entrez/query.fcgi?cmd=Retrieve&db=Nucleotide&list_uids=5514627&dopt=GenBank&RID=NB61GNHA01N&log$=nucltop&blast_rank=47)) | 93 |  | Uncultured eukaryote ([EF100310.1](http://www.ncbi.nlm.nih.gov/entrez/query.fcgi?cmd=Retrieve&db=Nucleotide&list_uids=118420157&dopt=GenBank&RID=NB61GNHA01N&log$=nucltop&blast_rank=1)) | 91 |  |  | - | 1 | - | T23_11 |
| **Rhizaria** |  |  |  |  |  |  |  |  |  |  |  |
| Cercozoa | Protaspis obliqua (FJ824122) | 87 |  | Uncultured cercozoan (AY620309) |  |  |  | 1,6 | - | - | T0_10 |
|  | Protaspis sp. ([FJ824125.1](http://www.ncbi.nlm.nih.gov/entrez/query.fcgi?cmd=Retrieve&db=Nucleotide&list_uids=225794704&dopt=GenBank&RID=NB95VVTG01S&log$=nucltop&blast_rank=3)) | 98 |  | Protaspis sp. ([FJ824125.1](http://www.ncbi.nlm.nih.gov/entrez/query.fcgi?cmd=Retrieve&db=Nucleotide&list_uids=225794704&dopt=GenBank&RID=NB95VVTG01S&log$=nucltop&blast_rank=3)) | 98 |  |  | - | - | 2,35 | T66_18 |
|  | Cercozoa sp. ([FJ824126.1](http://www.ncbi.nlm.nih.gov/entrez/query.fcgi?cmd=Retrieve&db=Nucleotide&list_uids=225794705&dopt=GenBank&RID=NB61GNHA01N&log$=nucltop&blast_rank=9)) | 84-93 |  | Cercozoa sp. ([FJ824126.1](http://www.ncbi.nlm.nih.gov/entrez/query.fcgi?cmd=Retrieve&db=Nucleotide&list_uids=225794705&dopt=GenBank&RID=NB61GNHA01N&log$=nucltop&blast_rank=9)) | 84-93 |  |  | - | 1,9 | - | T23_5,19 |
|  | Cercozoa sp. ([FJ824126.1](http://www.ncbi.nlm.nih.gov/entrez/query.fcgi?cmd=Retrieve&db=Nucleotide&list_uids=225794705&dopt=GenBank&RID=NB61GNHA01N&log$=nucltop&blast_rank=9)) | 92-95 |  | Uncultured marine cercozoan ([FN598348.1](http://www.ncbi.nlm.nih.gov/entrez/query.fcgi?cmd=Retrieve&db=Nucleotide&list_uids=282952415&dopt=GenBank&RID=NB61GNHA01N&log$=nucltop&blast_rank=1)) | 98-99 |  |  | - | 1,9 | 3,5 | T23_3; T66_15,23 |
|  | Massisteria marina ([AF174370.1](http://www.ncbi.nlm.nih.gov/entrez/query.fcgi?cmd=Retrieve&db=Nucleotide&list_uids=8248042&dopt=GenBank&RID=NB61GNHA01N&log$=nucltop&blast_rank=3)) | 98 |  | Massisteria marina ([AF174370.1](http://www.ncbi.nlm.nih.gov/entrez/query.fcgi?cmd=Retrieve&db=Nucleotide&list_uids=8248042&dopt=GenBank&RID=NB61GNHA01N&log$=nucltop&blast_rank=3)) | 98 |  |  | - | 1 | - | T23_20 |
| Foraminifera | Ammonia beccarii (U07937) | 85 |  | Uncultured eukaryote (EF100335) | 86-87 |  |  | 2,5 | 2,9 | 7,1 | T0_22; T23_9; T66_14 |
| **Amoebozoa** |  |  |  |  |  |  |  |  |  |  |  |
| Centramoebida | Corallomyxa sp. (EF514503) | 97 |  | Corallomyxa sp. (EF514503) | 97 |  |  | 0,8 | - | - | T0_36 |
| **Viridiplantae** |  |  |  |  |  |  |  |  |  |  |  |
| Chlorophyta | Nannochloris sp. (AB183585) | 99 |  | Nannochloris sp. (AB183585) | 99 |  |  | 0,8 | 1 | - | T0_44;T23_13 |
|  | Pyramimonas aureus([AB052289.1](http://www.ncbi.nlm.nih.gov/entrez/query.fcgi?cmd=Retrieve&db=Nucleotide&list_uids=39573521&dopt=GenBank&RID=NB95VVTG01S&log$=nucltop&blast_rank=1)) | 98 |  | Pyramimonas aureus([AB052289.1](http://www.ncbi.nlm.nih.gov/entrez/query.fcgi?cmd=Retrieve&db=Nucleotide&list_uids=39573521&dopt=GenBank&RID=NB95VVTG01S&log$=nucltop&blast_rank=1)) | 98 |  |  | - | - | 1,2 | T66_25 |
| Streptophyta | Carallia brachiata ([FJ707525.1](http://www.ncbi.nlm.nih.gov/entrez/query.fcgi?cmd=Retrieve&db=Nucleotide&list_uids=257853458&dopt=GenBank&RID=NB95VVTG01S&log$=nucltop&blast_rank=1)) | 99 |  | Carallia brachiata ([FJ707525.1](http://www.ncbi.nlm.nih.gov/entrez/query.fcgi?cmd=Retrieve&db=Nucleotide&list_uids=257853458&dopt=GenBank&RID=NB95VVTG01S&log$=nucltop&blast_rank=1)) | 99 |  |  | - | - | 3,5 | T66_2 |

*T0 0%, samples from T0 without oil contamination; T23 2%, 23 days after 2% of oil contamination; T66 2%, 66 days after 2% of oil contamination. Yellow lines indicate the most promising targets to be used in oil biomonitoring.
