# Supplementary figures and images for: 18S rDNA Sequences from Microeukaryotes Reveal Oil Indicators in Mangrove Sediment
Source: PLoS One. 2010 Aug 26;5(8):e12437. doi: 10.1371/journal.pone.0012437 (PMC2928742; doi:10.1371/journal.pone.0012437)

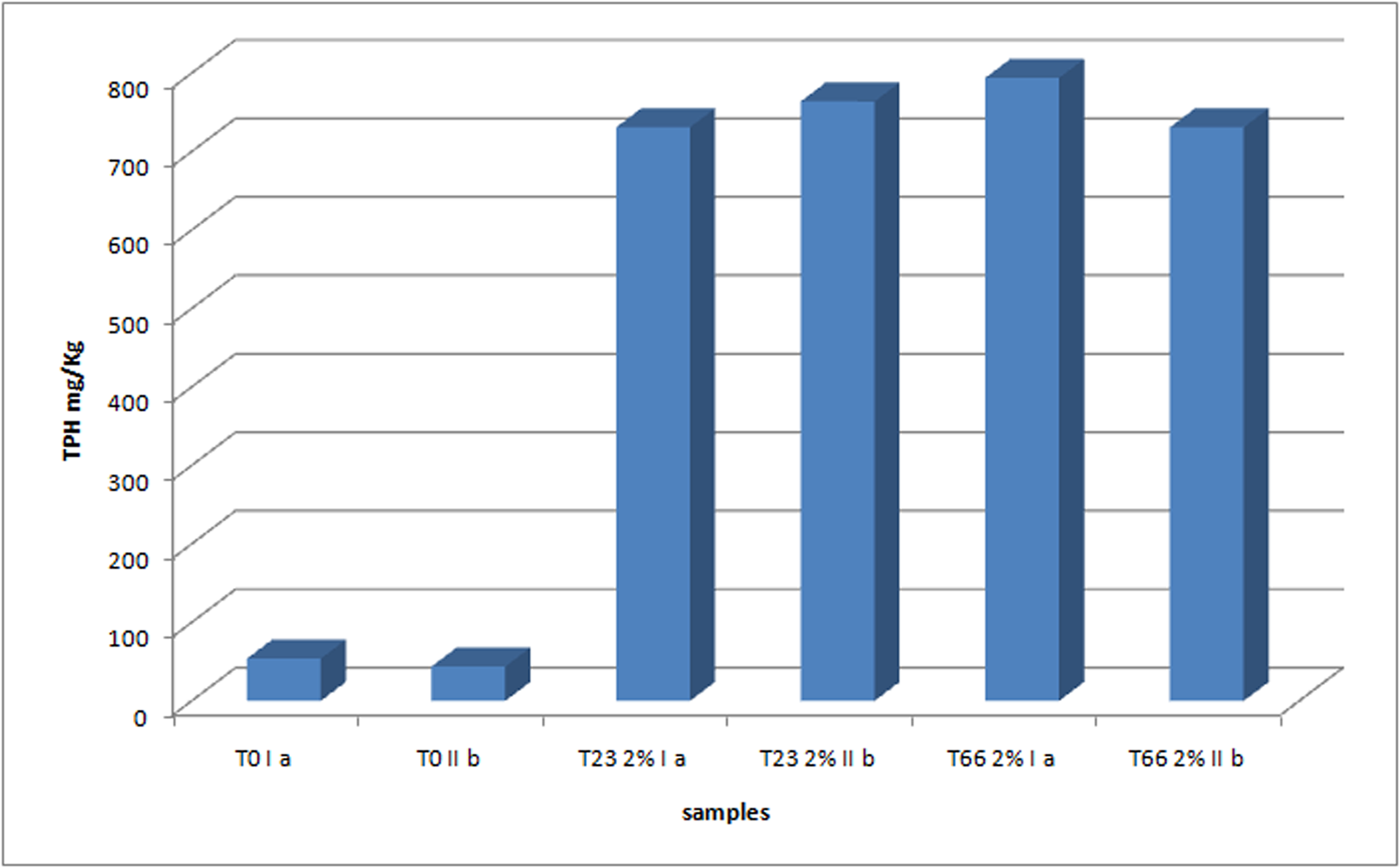

Supplement: Figure S1 — Total Petroleum Hydrocarbons (TPH) concentrations during experiment sampling. T0, without oil contamination; T23 2%, 23 days after 2% of oil contamination; T66, 66 days after 2% of oil contamination (duplicates). (9.47 MB TIF) [file pone.0012437.s002.tif]
